# Trends and Weekly Cycles in a Large Swiss Emergency Centre: A 10 Year Period at the University Hospital of Bern

**DOI:** 10.3390/ijerph14101239

**Published:** 2017-10-17

**Authors:** Christian T. Braun, Cornelia R. Gnägi, Jolanta Klukowska-Rötzler, Sufian S. Ahmad, Meret E. Ricklin, Aristomenis K. Exadaktylos

**Affiliations:** 1 Department of Emergency Medicine, Inselspital, University Hospital Bern, University of Bern, 3010 Bern, Switzerland; Christian.Braun@helios-kliniken.de (C.T.B.); cornelia.gnaegi@gmail.com (C.R.G.); meret.ricklin@gmail.com (M.E.R.); Aristomenis.Exadaktylos@insel.ch (A.K.E.); 2 Emergency Department and Rescue Medicine, Helios Klinikum Bad Saarow, 15526 Bad Saarow, Germany; 3 Department of Orthopedic Surgery, Inselspital, Bern University Hospital, 3010 Bern, Switzerland; sufian.ahmad@insel.ch

**Keywords:** University Emergency Centre (UNZ), general practitioner (GP)

## Abstract

Popular demand for high quality care has increased in recent years. This is also the case for medical services and support at all times of the day and night is nowadays required. During the last ten years, there has been a marked increase in the demands on hospital emergency hospitals, particularly in the Western industrialized countries. The present retrospective study investigates how the demands on a large Swiss university centre have changed over a period of 10 years. Patient numbers are differentiated by age, gender, nationality, weekday and mode of referral. A retrospective analysis was performed of the data of the patients admitted to the Emergency Centre of Bern University Medical Hospital (Inselspital) during the ten-year period from 2004 up to and including 2013 and who were treated as emergencies. A total of 264,272 patients were included in the study. It was shown that there was an uninterrupted annual increase from 23,555 patients in 2004 to 34,918 patients in 2013 (+48%). Most patients came to the Emergency Centre on Mondays, followed by Fridays. Because of the marked increase in life expectancy and the resulting demographic changes, there has been a marked increase in the number of older patients coming to the Emergency Centre for acute medical care. It was found that there were disproportionately high numbers of patients aged 20 to 49 years who were not Swiss citizens. In contrast, most patients over 60 were Swiss. In the coming years, emergency centres will have to adapt to the continued increase in patient numbers. This trend will continue, so that it is essential to consider the sociodemographic structure of a region when planning the availability of emergency medical care.

## 1. Introduction

Popular demand for high quality care has increased in recent years. This is also the case for medical services and support at all times of the day and night is nowadays required. Within the Swiss health system, this service is now provided by emergency practices, telephone medical hotlines and hospital emergency centres working 24 h a day, 365 days a year.

During the last ten years, the demands on hospital emergency services have greatly increased, particularly in Western industrialized countries [1]. According to statistics from the USA, the number of patient visits to emergency centres has increased by 5% to 11% annually [2,3]. The German Interdisciplinary Society for Emergency Admissions reported that the number of emergency patients admitted to German hospitals increased by 16.6% between 2005 and 2008 [4]. In 2009, the Austrian Medical Association reported that the number of outpatients in Austrian hospitals had increased by 55% between 1997 and 2007 [5,6,7].

Over the past 50 years, few European states have experienced as much demographic growth as Switzerland. Since 1960, the population has exploded, from just over five million inhabitants to over eight million (8,417,700 in 2016)—a boom mostly driven by immigration from Europe. In 2016 the natural increase was positive, as the number of births exceeded the number of deaths by 17,787. Due to external migration, the population increased by 79,166. The gender ratio of the total population was 0.968 (968 males per 1000 females) which is lower than global gender ratio (1016 males to 1000 females in 2016). As of the beginning of 2017 a Swiss population had the following age distribution: under 15 years old (15.2%), 15–64 years old (67.8%) and 65+ (17%) [8,9]. In the Canton of Bern, the ratio of people aged 64 or older to those aged 29–64 increased annually from 29.6 in 2007 to 33.5 in 2016 [10].

Because of the marked increase in life expectancy and the abovementioned demographic changes, the number of older patients transported by the ambulance service or coming to the Emergency Centre for acute medical care has also increased greatly [11,12]. Classical emergency centres are designed to provide the best possible medical care as quickly as possible. However, most problems that cause older people to come to emergencies are linked to chronic and often complex diseases and treating these patients requires more time and resources. The aging population of developed countries, and also in Switzerland, continues to have a large and disproportionate effect on emergency department operations.

Our literature search showed that there were no figures or analyses of patient numbers by nationality, age and mode of referral, therefore the present retrospective study examines changes in the numbers of patients treated by emergency centres and differentiated by age, gender, nationality, weekday and mode of referral. Our study is based on the admissions to a large Swiss university centre and covers a period of 10 years.

## 2. Materials and Methods

A retrospective analysis was performed of the data of the patients admitted to the University Emergency Centre (UNZ) of Bern University Medical Hospital (Inselspital) during the 10 year period from 2004 up to and including 2013 and who were treated as emergencies. We employed the clinical programs Qualicare (Qualidoc, Trimbach, Switzerland) for the period from 1 January 2004 to 30 May 2012 and E.care (E.care BVBA, Turnhout, Belgium) for the period from 1 June 2012 to 31 December 2013. The Swiss Federal Office for Statistics provided data from the Foreign Population Structure and Migration Statistics (PETRA, 2003–2009) and Population and Households Statistics (STATPOP, 2010–2013) databases, in order to allow a comparison between the patients and registered Swiss citizens with respect to age and nationality. Data processing and all calculations were performed with Microsoft Excel (Microsoft, Redmond, WA, USA). The data were first cleaned in accordance with the inclusion and exclusion criteria for the original patient pool. The inclusion criteria were fulfilled by all patients aged over 16 years whose gender could be unambiguously defined and whose definitive nationality was given. Data from 277,223 patients were exported from the both hospital information systems. A total of 12,951 (4.67%) patients were excluded from the study. In addition, 8460 (3.05%) duplicated patient entries and test patients were sorted out, together with 3295 (1.19%) patients without documented nationality, 48 (0.02%) patients without defined gender and all patients aged under 16 years (1148 patients, 0.41%). This left 264,272 patients for further analysis.

The patient numbers during the period of observation were analysed with respect to age, gender and nationality. We were also interested in the mode of referral and the weekday of the presentation. The patients were split into three groups on the basis of their nationality: Swiss citizens, European Economic Area (EEA) citizens and non-EEA citizens. We also investigated the mode of referral to the emergency centre and the identity of the referrer. The referral was subdivided into eight categories: Ambulance Service, External Hospital, general practitioner (GP), Internal Referral, Army, Police, Psychiatrist/Psychiatry, Rega (Swiss Air Rescue), Rega (Repatriation), Self-Referral and Miscellaneous.

## 3. Results

### 3.1. Patient Numbers

**Figure 1:**
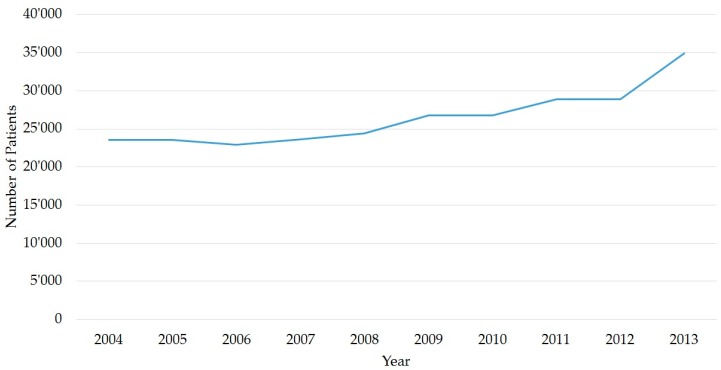
Numbers of Patients. Absolute numbers of patients per year presenting to the Emergency Centre (UNZ), by year (*N* = 264,272 patients).

After exclusion, mostly because of duplicate patients, our data contained 264,272 patients for further analysis. We observed a steady annual increase, from 23,555 patients in 2004, to 26,802 patients in 2009 to 34,918 patients in 2013 (Figure 1). Between 2004 and 2007, this corresponded to a mean value of ca. 64 patients per day. From 2008, annual patient numbers increased, up to a maximum of 96 patients per day in 2013 (Table 1).

### 3.2. Gender

For the whole period, a mean of 42% female and 58% male patients were treated. The gender distribution was constant over the 10 years. If the individual years are examined, it is evident that this percentage was stable and hardly changed during the individual years.

### 3.3. Age Distribution

**Figure 2:**
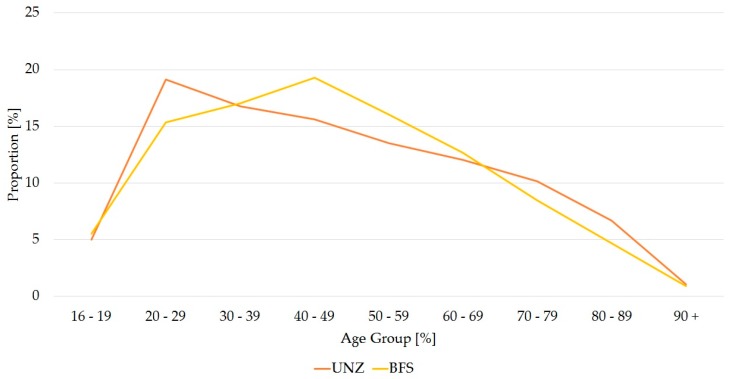
Percentage distribution of the age groups (years) of the permanent Swiss population, compared with Emergency Centre patients (permanent Swiss population 2004–2013, *n* = 53,072,512 persons (UNZ); Emergency Centre patients, *n* = 264,272 persons (BFS).

As regards the age distribution, the values were markedly higher for younger patients, with a peak at 26 years of age. The age group with the most patients was for the 20–29 year olds, with 19% of the total (50,636 patients). From the peak for the 26 year olds, the mean age decreased continuously up to the 80 year olds and then dropped sharply for the over 80 years old, who made up 8% of the total patient population (Table 2). Up to age 70, the percentage age distribution of the patients in the UNZ was slightly displaced to the left in comparison with the distribution in the general Swiss population. There was a slight right displacement for patients aged over 70 (Figure 2). The peak for the total Swiss population was at 45 years and generally in the 40–49 year old group. As with the emergency patients, the total numbers in the Swiss population decreased continuously with age. Patients aged over 90 years made up only 1% of the total population (Figure 2).

**Figure 3:**
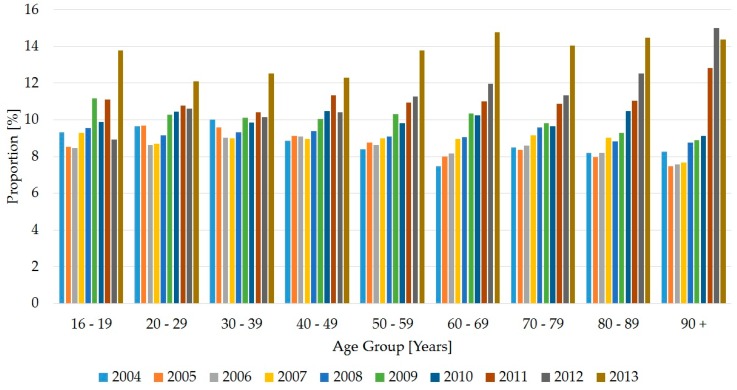
Percentage distribution of age groups (years), by year (*N* = 264,272 persons).

The different age groups increased over time in a very similar manner (Table 3). The greatest increase was from 2012 to 2013 and was observed in each age group. The only exception was with patients aged over 90, who decreased from the previous year. On the other hand, the relative increase over the 10 years was greatest for patients aged over 90—from 8% to 14% (Figure 3).

On Mondays, the values for the different age groups were very similar: 14.3–15.6%. On Fridays, relatively more older patients presented to the UNZ (Table 4). Patients aged 70–79 years old then made up the largest individual group, with 16%. The inverse relationship was found on Sundays; most patients were aged 16–49 years; 17% were in the 16–19 years old, 16% aged 20–29 years and 14% 40–49 years old.

### 3.4. Referrals

**Figure 4:**
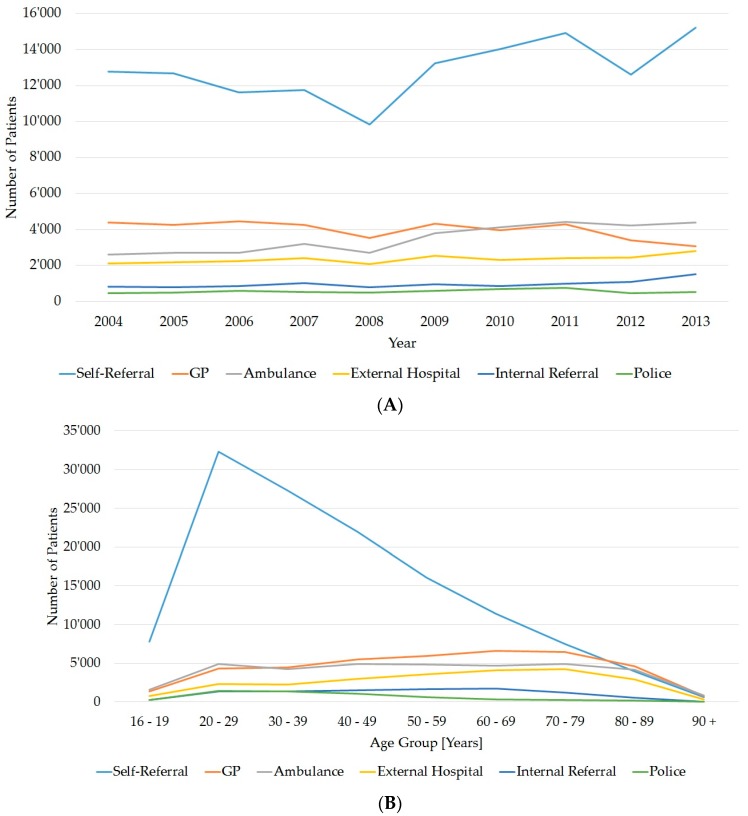

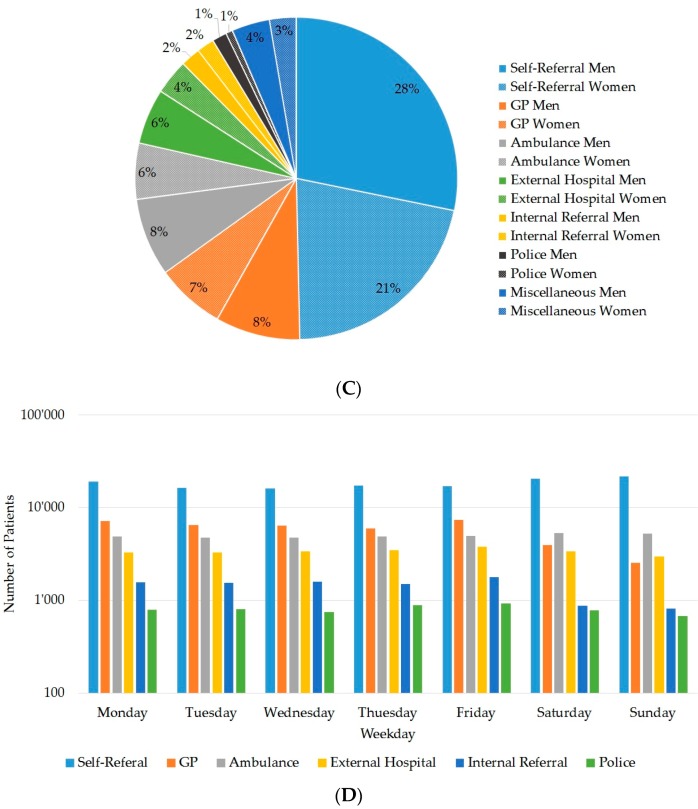
(**A**) Numbers of patients for each year, by mode of referral (*N* = 242,310 patients, 21,962 patients without mode of referral); (**B**) Absolute numbers of patients for each mode of referral, by age group (*N* = 242,310 patients, 21,962 patients without mode of referral); (**C**) Percentage distribution of each mode of referral, by gender (women *n* = 112,062; men *n* = 152,210); (**D**) Absolute patient numbers, by weekday and mode of referral (*N* = 242,310 patients, 21,962 patients without mode of referral). GP: general practitioner.

Over the 10 years, the largest group of patients were self-referrers (more than 48%). This group also made the greatest contribution to the increase in the numbers of emergency patients (Figure 4A). The next largest group were patients referred by a GP (15%), followed by those referred by the ambulance service (13%), patients referred from other hospitals (9%), patients from other departments in the Inselspital (3%) or patients accompanied by the police (2%), who normally came directly from prison. Most self-referrers were aged 20–29 years (Figure 4B). All other modes of referral were evenly distributed throughout the age groups. There were no significant differences in gender aspects concerning the self-referrers, the patients referred by a GP or from other hospitals (Figure 4C).

### 3.5. Referral by Weekday

Most self-referrals occurred during the weekend or on Mondays. GPs most frequently referred on Mondays and Fridays and internal departments on Fridays (Figure 4D). Referrals by the ambulance service, external hospitals or the police were evenly distributed throughout the week.

### 3.6. Population Groups

**Figure 5:**
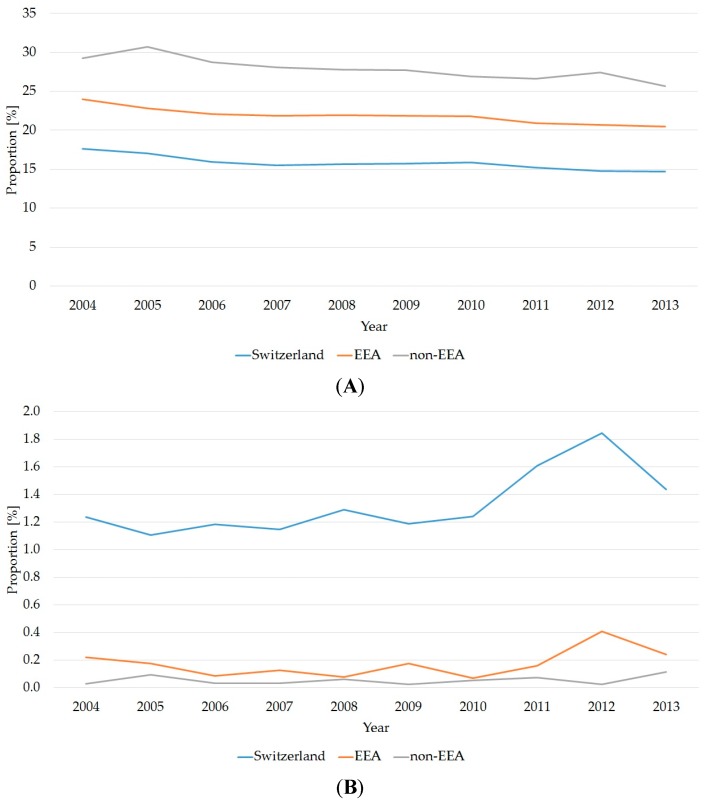
Nationality and different age segments. (**A**) Percentage changes within the patient population of 20–23 year olds over 10 years (Switzerland = 35,638 patients, EEA = 5369 patients, non-EEA = 9620 patients); (**B**) Percentage changes within the patient population aged over 90 years over 10 years (Switzerland = 2710 patients, EEA = 50 patients, non-EEA = 20 patients). EEA: European Economic Area.

In comparison to Swiss citizens, the EEA and non-EEA populations were disproportionately represented in the younger age segments—i.e., between 20 and 49 years (Figure 5A). This ratio was inverted in patients aged 60 or more and the relative number of Swiss citizens increased with increasing age. This is most marked with Swiss patients aged over 90, where the Swiss patients were much older.

## 4. Discussion

Today, there are more old people than at any other point in history. This is due to several factors including decreasing fertility rates, better public health measures, and advances in the field of medicine [13]. During the last decades Switzerland has had one of the largest growth rates in Western Europe and the population has more than tripled since 1860. Cities and urban communities have grown dynamically over the past decade, according to the Association of Swiss Cities. In 1900, there were 76 young people under age 20, and 10 persons 65 years or older for every 100 persons of working age (ages 20–64). This ratio has changed significantly: in 2013, there were only 33 young people, and 28 persons 65 or older for every 100 of working age. Thus, the old-age dependency ratio has almost tripled while the youth-dependency ratio has halved [14].

In previous research projects described that hospital admission depending of the weekday was associated with increased mortality and greater lengths of stay. Some studies attribute worse weekend outcomes, referred to as the “weekend effect” to lower hospital staffing levels and service availability [15].

Our study presents descriptive data for a large Swiss Emergency Centre in Bern and focuses on differences between weekend and weekday admissions in the period 2004–2013 for patient, hospital, and stay characteristics. This information provides an overview of differences between patients admitted on workdays and in the weekends.

In the last 10 years patient numbers have increased at this centre. Similar results have been obtained for the last 20 years in the USA, Canada and Spain [16,17,18,19]. Most patients in our study came on the weekdays immediately before and after the weekend, were young and often came independently. These patients presumably exploited the possibility of having an appointment with a doctor in an emergency centre before the coming weekend without having to fix an appointment. On the other hand, some delayed visiting the emergency centre till Monday, presumably because these patients expected that routine work in the emergency centre would be reduced during the weekend. This was also found in another study, in which most consultations took place on Mondays [20]. In comparison with an appointment with a GP, a visit to the emergency centre presumably has the advantage that no appointment was necessary, as was confirmed in another study [21].

Other possible reason may be that the patients who refer themselves—who are mostly young—have little or no connection to outpatient medical care and therefore have no GP whom they could have consulted rapidly. They also see the emergency centre as the best way of solving their health problems. This at least corresponds to the observations in other studies [22,23,24]. In the present study and in one other study, most patients were male [25]; in contrast, there were slightly more female patients in a Scottish study, so that there is no unambiguous gender trend in emergency admissions [26]. In the age distribution, there is a marked increase in young patients, with a peak for the 26-year olds. The greatest numbers are in the 20–29 year age group. Studies from the USA demonstrate that there have been increases in the total number of young patients and patient visits in general to hospital emergency centres. Moreover, there have been increases in the numbers of geriatric patients [27].

As in our study, there was a marked increase in the number of over 65-year olds admitted to emergency centres. The greatest percentage increase was in the group of over 85-year olds. In general, in recent years, approximately 20% of all patients requiring help from emergency centres have been older [27,28]. At the moment, more than 25% of patients in the emergency centre of the Inselspital are older than 65 years. There have also been marked increases in the numbers of very old patients above 80 or 95 years of age [29]. Over the 10-year observation period, there has been a marked increase in self-referrals. There are now 50% of self-referrals, so that this corresponds to a large proportion of the increase in patients [26]. In our study, the self-referrers tended to be young. The reason may be that young patients tend not to have a fixed GP [30].

Particularly with younger populations, especially the non-EEA citizens [31], with their predominantly young populations, it should be possible to improve patient flow in emergencies, by establishing alternative sites of access, such as GP health care centres attached to emergency centres. This could help patients without a Swiss passport become better integrated in the GP system [30,31,32], as patients from non-EEA states are apparently the group which is least well integrated into the system [19,33].

Particularly in large cities, a large proportion of emergency patients are immigrants. These patients have not been properly studied in health care research and little is known about their needs. Studies from the 1990s have shown that immigrants can only be provided with adequate health care if their specific needs and histories are born in mind. This is often neglected in emergency centres [34,35,36,37,38,39]. 

Some articles report that ethnic minorities place high demands on emergency services, as was found in our study [30,31]. It should however be emphasised that this mainly applied to younger patients aged up to 60. Most older patients were Swiss.

## 5. Conclusions

In the coming years, emergency centres will have to adapt to a continued increase in patient numbers. For the organisation and optimisation of patient care in emergency centres, it is important to know that the weekdays with the highest patient numbers are Monday—with mostly younger patients—and Friday—with mostly older patients. Most patients refer themselves. The number of self-referrers is also continuing to climb; most self-referrers are younger and are not Swiss. It was also found that emergency centres must prepare for a marked increase in the number of geriatric patients. In particular, the group of very old patients of over 90 will increase. For this reason, emergency centres must prepare themselves to provide adequate care to this group of patients.

These trends are expected to continue, so that it is essential to consider the sociodemographic structure of a region when planning the availability of emergency medical care. Future studies should attempt to analyse the main health problems of each group of patients, e.g., the young self-referrers or the geriatric patients, in an attempt to improve the planning of available services. 

### Limitations

The principle limitation of this study is that it is a retrospective data analysis.

A further limitation is that the population data that is used to compare the results of the investigation in the Emergency Department is not directly related to the population area of the hospital. The data of the Federal Office of Statistics (BFS) on the population up to 2009 was administered by the PETRA synthesis statistics and is based on the following official registers or administrative data: Central Register of Foreigners of the Federal Office for Migration, Register (ORDIPRO) of the Federal Department of Foreign Affairs, Register of the Asylum Section (AUPER) of the Federal Office for Migration, as well as the results of the statistical study on natural migration of populations (BEVNAT). From 2010, the data of the Federal Office of Statistics BFS on population were collected by STATPOP (part of the statistical study in the context of the new annual census system). This switch in 2010 influenced the evaluation and graphical presentation of the data and led to a discontinuous jump in the curve for the resident foreign population.

## Figures and Tables

**Table 1:** Mean daily number of patients presenting to the Emergency Centre by year (*N* = 264,272 patients, ±SD = 9.68).

Year	Patients/Day
2004	64
2005	65
2006	63
2007	65
2008	67
2009	73
2010	73
2011	79
2012	79
2013	96

**Table 2:** Absolute numbers of patients over ten years, by age group (years).

Age Group	Number of Patients	EC	BFS
16–19	13,269	5%	6%
20–29	50,636	19%	15%
30–39	44,333	17%	17%
40–49	41,269	16%	19%
50–59	35,771	14%	16%
60–69	31,781	12%	13%
70–79	26,774	10%	8%
80–89	17,658	7%	5%
90+	2781	1%	1%

EC: Emergency Centre; BFS: Federal Office of Statistics.

**Table 3:** Absolute patient numbers, by age group (years) and years (*N* = 264,272 persons).

Age (Years)	2004	2005	2006	2007	2008	2009	2010	2011	2012	2013
16–19	1237	1130	1124	1233	1267	1481	1311	1476	1183	1827
20–29	4890	4911	4375	4403	4630	5200	5287	5452	5366	6122
30–39	4444	4250	4004	3990	4129	4478	4364	4623	4500	5551
40–49	3654	3766	3756	3696	3876	4142	4326	4681	4299	5073
50–59	3005	3130	3090	3212	3249	3690	3513	3919	4031	4932
60–69	2372	2539	2601	2846	2882	3293	3253	3494	3803	4698
70–79	2275	2244	2302	2452	2570	2633	2585	2914	3039	3760
80–89	1448	1409	1446	1594	1560	1638	1849	1948	2211	2555
90+	230	208	211	213	244	247	254	357	417	400

**Table 4:** Percentage distribution of age group, by weekday of visit (*N* = 264,272 persons).

Weekday	16–19	20–29	30–39	40–49	50–59	60–69	70–79	80–89	90+
Monday	15%	15%	15%	15%	16%	15%	16%	15%	14%
Tuesday	13%	13%	14%	14%	14%	14%	14%	14%	14%
Wednesday	13%	13%	13%	14%	14%	14%	14%	14%	14%
Thursday	13%	13%	14%	14%	14%	14%	14%	14%	15%
Friday	14%	14%	14%	14%	15%	16%	16%	16%	16%
Saturday	16%	15%	15%	14%	14%	14%	13%	14%	14%
Sunday	17%	16%	15%	14%	13%	12%	12%	12%	13%
